# Does winter cold really limit the dengue vector *Aedes aegypti* in Europe?

**DOI:** 10.1186/s13071-020-04054-w

**Published:** 2020-04-07

**Authors:** Isabelle M. Kramer, Aljoscha Kreß, Doris Klingelhöfer, Christian Scherer, Parbati Phuyal, Ulrich Kuch, Bodo Ahrens, David A. Groneberg, Meghnath Dhimal, Ruth Müller

**Affiliations:** 1 Institute of Occupational, Social and Environmental Medicine, Goethe University, Frankfurt am Main, Germany; 2 Institute for Atmospheric and Environmental Sciences, Goethe University, Frankfurt am Main, Germany; 3 Nepal Health Research Council, Kathmandu, Nepal; 4 Unit Entomology, Institute of Tropical Medicine, Antwerp, Belgium

**Keywords:** Cold hardiness, Distribution limits, Cold tolerance, Overwintering, Phenotypic plasticity, Sub-zero exposure, Winter survival

## Abstract

**Background:**

*Aedes aegypti* is a potential vector for several arboviruses including dengue and Zika viruses. The species seems to be restricted to subtropical/tropical habitats and has difficulties in establishing permanent populations in southern Europe, probably due to constraints during the winter season. The aim of this study was to systematically analyze the cold tolerance (CT) of *Ae. aegypti* in its most cold-resistant life stage, the eggs.

**Methods:**

The CT of *Ae. aegypti* eggs was compared with that of *Ae. albopictus* which is well established in large parts of Europe. By systematically studying the literature (meta-analysis), we recognized that CT has been rarely tested in *Ae. aegypti* eggs, but eggs can survive at zero and sub-zero temperatures for certain exposure periods. To overcome potential bias from experimental differences between studies, we then conducted species comparisons using a harmonized high-resolution CT measuring method. From subtropical populations of the same origin, the survival (hatching in %) and emergence of adults of both species were measured after zero and sub-zero temperature exposures for up to 9 days (3 °C, 0 °C and − 2 °C: ≤ 9 days; − 6 °C: ≤ 2 days).

**Results:**

Our data show that *Ae. aegypti* eggs can survive low and sub-zero temperatures for a short time period similar to or even better than those of *Ae. albopictus*. Moreover, after short sub-zero exposures of eggs of both species, individuals still developed into viable adults (*Ae. aegypti*: 3 adults emerged after 6 days at − 2 °C, *Ae. albopictus*: 1 adult emerged after 1 day at − 6 °C).

**Conclusions:**

Thus, both the literature and the present experimental data indicate that a cold winter may not be the preventing factor for the re-establishment of the dengue vector *Ae. aegypti* in southern Europe.
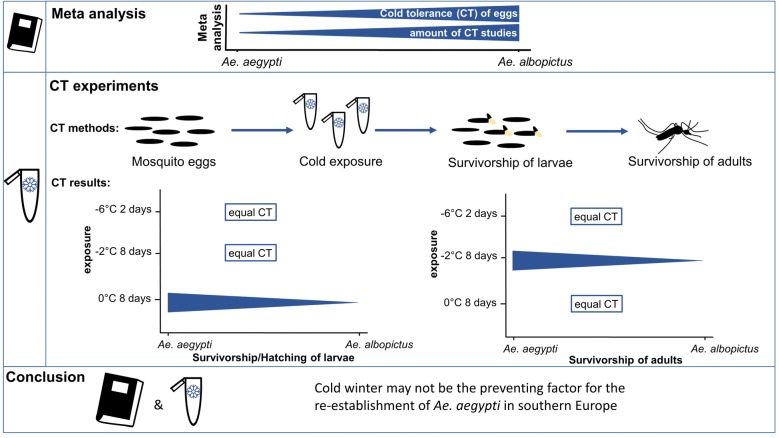

## Background

*Aedes aegypti* is the main vector for several arboviruses including dengue, chikungunya, yellow fever and Zika viruses [1]. A re-invasion of *Ae. aegypti* in southern Europe could become a major threat for the European public health systems. *Ae. aegypti* (Linnaeus, 1762), originally an African mosquito species, had already been present in Europe in the early 20th century when it spread from the Portuguese Atlantic coast to the Black Sea [2]. According to one hypothesis, the species never established, disappeared in winter and was transported to Europe again in spring every year. After the 1950s, *Ae. aegypti* disappeared in Europe due to malaria vector control campaigns using DDT (dichlorodiphenyltrichloroethane) indoor treatments, management of urban water collections, and possibly harsher winter climate conditions [3]. Between 1960 and 2000 only a few sporadic records of *Ae. aegypti* occurrence were reported from Italy, Israel and Turkey [2]. The species established on Madeira in 2004 [4] and it is known that it started to re-populate the northern coast of the Black Sea in 2007 [5]. In 2010, *Ae. aegypti* was for the first time detected in the Netherlands. However, control measures with adulticides and larvicides have been effective in this country [6]. By comparison, *Aedes* (*Stegomyia*) *albopictus* (Skuse, 1894) spread in the last 30–40 years from East Asia and islands of the western Pacific and Indian Ocean (subtropical/tropical areas) to all continents except Antarctica [7, 8]. In Europe, *Ae. albopictus* has been reported in 26 countries and is established in 20 of these countries [9, 10]. The massive spread of *Ae. albopictus* and the establishment of permanent populations especially in northern Europe was possibly caused by the strong ecological plasticity of the species which allows for its rapid adaptation to different kinds of habitats and even for its survival under mild winter conditions [11, 12]. Sub-zero temperatures affect *Ae. albopictus* depending on the origin of populations (temperate, subtropical or tropical; [12]) and photoperiod (i.e. diapausing or non-diapausing eggs; [13–16]). In temperate regions, *Ae. albopictus* can adapt to cold winters by producing dormant or so-called diapausing eggs [12]. *Aedes aegypti* lacks diapause and thus seems to be more restricted to subtropical and tropical habitats. Therefore, it has been assumed that *Ae. aegypti* has difficulties to establish permanent populations in Europe that can overwinter under the prevailing climatic conditions. In general, the mechanism of the *Aedes* larvae to survive within the eggshell at low temperatures is defined as the cold hardiness of eggs (reviewed in [13]). Underlying mechanisms of CT in *Ae. albopictus* eggs are reviewed by Kreß et al. [17]. Several cold tolerance (CT) studies with *Ae. albopictus* eggs [13, 15, 17–30] and *Ae. aegypti* eggs [20, 22, 27, 29–36] have already been conducted. Within this approach, CT of South Asian populations of the same origin of both species was evaluated for the first time.

The aim of this study is to compare the CT of the eggs of *Ae. aegypti* and *Ae. albopictus* by systematically reviewing the respective literature and conducting a meta-analysis, and by experimentally testing their eggs in a new comparative set-up using a high-resolution CT measuring method [17]. Taking the distribution of both species into account, we hypothesized that *Ae. albopictus* is in general more cold-hardy than *Ae. aegypti.*

## Methods

### Meta-analysis

A meta-analysis of the published CT data of *Ae. aegypti* and *Ae. albopictus* was conducted in accordance with the PRISMA guidelines (see also Additional file 1). All databases indexed in Web of Science (WoS) were searched for articles published until the 25th of October 2019 including Web of Science Core Collection, Biological Abstracts, BIOSIS Citation Index, Current Contents Connect, Data Citation Index, Derwent Innovations Index, KCI-Korean Journal Database, Medline, Russian Science Citation Index, SciELO Citation Index and Zoological Record. Only English language publications were included. A WoS topic search that included title, abstract and keywords was applied including the following terms: (i) species: *Aedes* AND *albopictus* OR *aegypti*; (ii) life stage of CT testing: egg; (iii) definition of CT: cold tolerance, cold hardiness, cold resistance, overwinter, sub-zero exposure.

In the meta-analysis, we included only studies that complied with these inclusion criteria and contained data of temperature, exposure time and hatching success defined as survivorship after sub-zero exposure. Only studies referring to zero and subzero temperatures (≤ 0 °C) were used for further analysis. The references of the retrieved articles were also checked for relevant articles; this resulted in the identification of six additional articles that fulfilled the inclusion criteria. Out of the total of 93 retrieved articles matching our inclusion criteria, 22 articles remained relevant for the meta-analysis. One matching article was excluded because it was not accessible [37]. Some data points had to be excluded either due to an incorrect labelling of graphs by authors or missing exact data on minimum temperature or survivorship [20, 31, 33, 34, 36].

The following data parameters were extracted and captured in table format: species, test temperatures, exposure time, rearing conditions (diapause or non-diapause), origin of populations tested, climatic zones of the origin (tropics and temperate (subtropics and tropics were summarized as tropics)), generation tested, survivorship, mortality, hatching (positive or negative), acclimation phase (presence, absence, and differences), type of study (laboratory or field), replicates (presence or absence), controls (presence or absence), number of eggs tested, rearing to life stage (larvae, pupae, adult), bleaching of unhatched eggs (yes or no), comment (if necessary regarding the extracted data), author, year, title, origin of publication, and limitations of study. If original data was needed for correct data extraction, corresponding authors provided original data on request [17, 22].

A detailed description of the extracted data and differences between studies are provided in Additional file 2. Extracted and included data points are given in Additional file 2: Tables S1, S2, respectively. The data points that were excluded from the meta-analysis and their respective exclusion arguments are presented in Additional file 2: Table S3. If only information about survivorship was given, mortality was calculated and *vice versa*. If precise information about the start and end dates of experiments were missing, especially in field studies, the exposure time was estimated (detailed calculation in Additional file 2: Table S4). The minimum temperature in field studies was always used for analysis despite temperature deviations during the exposure time. Referring to the approach described in [30] it was possible to extract minimum temperature from the given exposure times from the website of the National Oceanic and Atmospheric Administration (https://w2.weather.gov/climate/xmacis.php?wfo=pah). If necessary, exact survivorship could be calculated from pixels of extracted figures (Additional file 2: Table S5). Survivorship, exposure time and temperature were analyzed by generating three-dimensional plots separated for both species as well as for both study types (laboratory or field) using RStudio Version 1.1.423 [38]. The proportion of data points tested at different temperature levels (in total 100%) and time points (in total 100%) and analyzed parameters (each parameter in total 100%) were analyzed for each species and type of study. Minimum, maximum and mean of tested exposure time and temperature were examined. As additional parameters for meta-analysis, the number of generations, the absence or presence of replicates and controls, (non)acclimation to different temperatures before sub-zero exposure, the bleaching of unhatched eggs after sub-zero experiment, and whether hatched larvae were reared to pupal stage or adulthood after sub-zero exposure were included. Furthermore, publication year, the country of origin of publications (based on the institutional affiliation of the first author) and the origin of the tested mosquito populations were examined.

### Cold tolerance experiment

The CT of eggs of two *Aedes* species from the same South Asian sampling site was assessed at cold temperatures (CT1: 3 °C (≤ 9 days), 0 °C (≤ 4 days)) and (sub)zero temperatures (CT2: 0 °C,  − 2 °C (≤ 8 days) and − 6 °C (≤ 2 days)). Therefore, first the eggs were cooled down in a stepwise manner until exposure temperatures were reached; secondly, the eggs were exposed to sub-zero temperature for a certain period of time; thirdly, the eggs were subsequently warmed up and hatching stimulus was provided. The CT of eggs was determined in each treatment by quantifying survivorship (number of hatched larvae and the percentage that survive to adult stage).

**Fig. 1:**
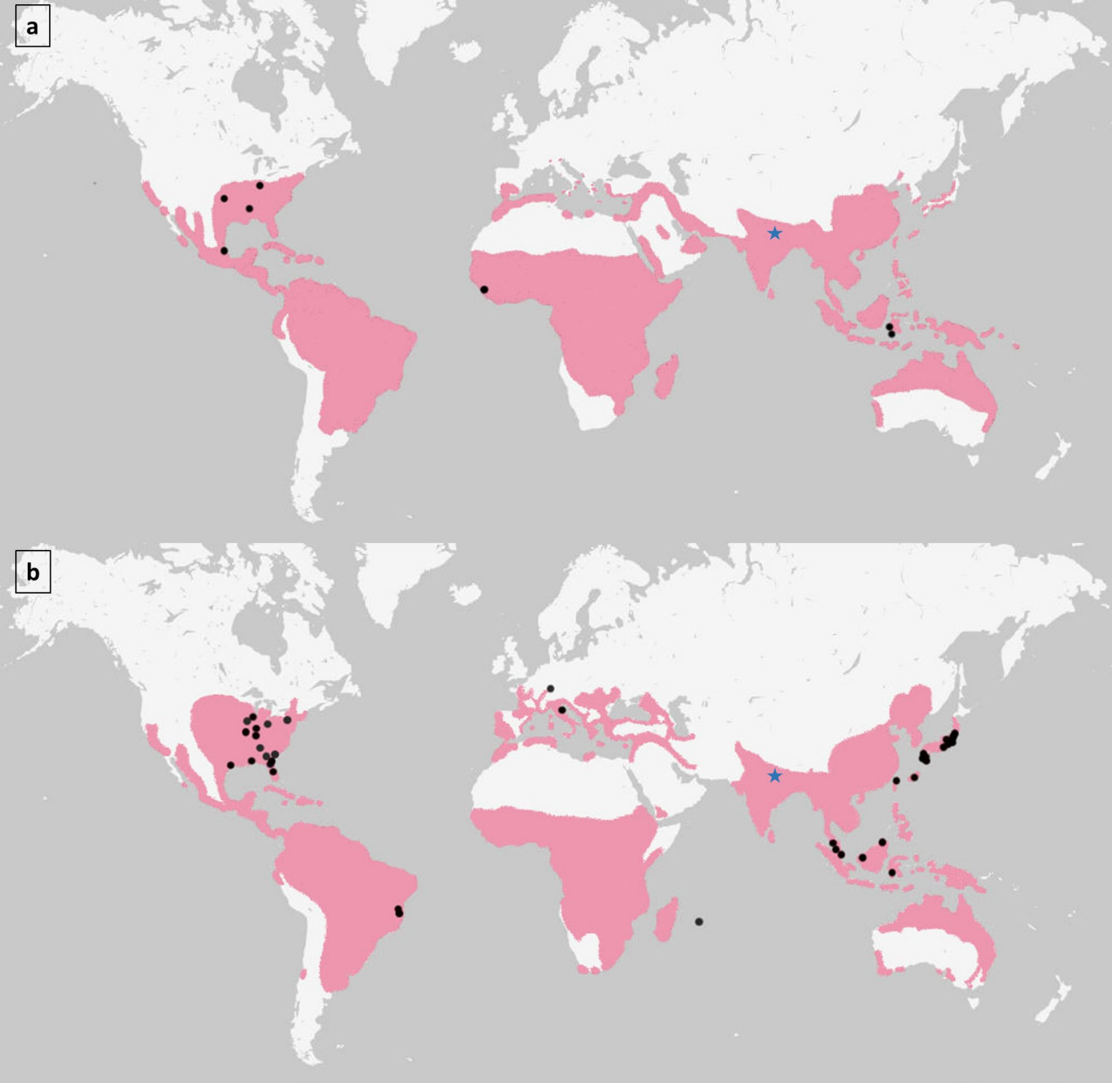
Origin of *Ae. aegypti* (**a**) and *Ae. albopictus* (**b**) populations with known cold tolerance (CT). The predicted distribution of the two species according to [47] is shaded in magenta. The origin of populations examined in CT studies included in the meta-analysis are marked with black dots. The sampling site of mosquitoes used for CT experiments in the present study (Chitwan, Nepal) is marked with a blue star (source of map: https://mapstyle.withgoogle.com/)

The experiments reported herein, are to the best of our knowledge, the first CT studies of *Ae. aegypti* and *Ae. albopictus* eggs from a South Asian population (Fig. 1). Eggs of both species were collected in Chitwan (27°39′03.8″N, 84°24′43.1″E), a lowland region of Nepal with a subtropical climate, in November 2017, shipped to the Department of Environmental Toxicology & Medical Entomology, Institute of Occupational, Social and Environmental Medicine, Goethe University Frankfurt am Main, Germany, and reared to adulthood under quarantine conditions [39]. For this purpose, eggs were placed into yeast solution and the hatching of larvae was induced [39]. L2 larvae were moved into 1 l vessels filled with water and were fed *ad libitum* with ground fish food (TetraMin flakes, Tetra, Germany; [40]). Pupae were individually moved into 2 ml tubes. After their emergence, water was removed and adult species identity determined under a stereo microscope with 50-fold magnification (SMZ-171; Motic^®^ Deutschland GmbH, Wetzlar, Germany). Adults of one species were released into a quarantine cage [39]. The breeding procedure (of F0 and F1) took place in a climate chamber with a light:dark cycle of 16:8 h (non-diapausing conditions) at 25 ± 0.44 °C and 80 ± 7.95% relative humidity. Females were fed with human blood approximately every second day [39]. Deposited eggs were counted on filter paper and stored at 25.1 ± 0.1 °C and 84.8 ± 3.3% relative humidity until the start of the experiments.

To determine CT, the high-resolution measuring method introduced by [17] was applied. In two CT experiments, eggs of *Ae. aegypti* and *Ae. albopictus* were exposed to cold temperatures (CT1) and (sub)zero temperatures (CT2) *versus* control conditions (25 °C) for 0.5 to 9 days (Table 1). Results of the CT1 experiment encouraged us to test the survivorship of *Ae. aegypti* eggs at sub-zero temperatures. In CT1, 2160 eggs of the F_1_ laboratory generation of *Ae. aegypti* were used. In CT2, 1600 eggs of *Ae. aegypti* and 1500 eggs of *Ae. albopictus* of the F_2_ laboratory generations were used. For each experimental treatment, 120 eggs were filled in 0.2 ml tubes in CT1, and 100 eggs in CT2, respectively. Those tubes were cooled in a 5 °C and 24 h stepwise manner from 25 °C downwards. In CT1, PCR cyclers (TProfessional Basic and T3 Thermocycler; Biometra GmbH, Jena, Germany) were programmed to hold constantly all temperatures until 0 °C. In CT2, PCR-cyclers were programmed to hold constantly all temperatures until 5 °C, respectively. The accuracy and function of PCR cyclers was checked using a laser temperature measurement device (Testo SE & Co.KGaA, Lenzkirch, Germany). In CT2, 0 °C samples were installed in a cold-water thermostat by placing 0.2 ml tubes in 50 ml tubes in the thermostat (RM 20 LAUDA, Dr R. Wobser GmbH & CO. KG, Lauda-Königshofen, Germany). A cooling box connected to a thermostat (Model F12-ED; Julabo GmbH, Seelbach, Germany) was used to carry out the − 2 °C exposure of eggs [17]. The temperature at the exact position of the egg tubes in the cooling box was measured daily using a data logger (HOBO UX100-011; Onset, Cape Cod- MA, USA). On average, a temperature of − 1.9 ± 0.2 °C was measured in the cooling box. It has to be noted, that short-term > 0 °C data resulting from the opening of the cooling box during handling were excluded from the analysis. The water/glycol tank of a thermostat (Model F12-ED; Julabo GmbH) was used for − 6 °C CT treatments. The temperature at the exact position of the egg tubes in the − 6 °C and 0 °C thermostat experiments was confirmed daily using a thermometer (Thermometer Deluxe, error rate 0.1 °C; Lucky Reptile, Waldkirch, Germany); no temperature variation over the experimental time was observed.

**Table 1:** Cold tolerance (CT) experiments, CT1 with *Ae. aegypti* eggs and CT2 with *Ae. aegypti* and *Ae. albopictus* eggs

CT experiment	Generation	Temperature (°C)	Exposure time (days)												
CT1: *Ae. aegypti*	F1	0	0.5	1	1.5	2	2.5	3	3.5	4					
		3		1		2		3		4	5	6	7		9
CT2: *Ae. aegypti*	F2	0		1		2				4				8	
		− 2		1		2		3		4^a^		6		8	
		− 6		1		2									
CT2: *Ae. albopictus*	F2	0		1		2				4		6		8	
		− 2		1		2		3		4		6		8	
		− 6		1		2									

^a^Technical error, two treatments instead of one

*Notes*: Experimental conditions including the laboratory generation of species, exposure temperature and exposure times (treatment, in days) are given. In parallel, two negative controls (NC) were run at 25 °C during each experiment

To induce hatching after the exposure of eggs to specific CT treatments (Table 1), eggs were placed into 12-well culture plates with ~ 10 eggs and 2 ml of hatching solution [39] per cavity and exposed at 25 °C to continuous light for 24 h, and then to a 16:8 h light:dark cycle for another six days (CT1) or five days (CT2). One well was regarded as one replicate per treatment (CT1: 10–12 replicates; CT2: 8–10 replicates). Each treatment represented 94–124 (CT1) and 70–106 (CT2) eggs, respectively.

Survivorship after sub-zero temperature exposure was determined using a stereo microscope with 50-fold magnification (SMZ-171, Motic^®^; Deutschland GmbH). The minimum requirement for survivorship was defined as the head of the larva being visible outside the chorion [17]. In order to close knowledge gaps in the literature, we also reared both species after sub-zero exposure in the CT2 experiment to the adult stage which is most relevant in a medical context. The sex of adult specimens was checked, and species identity verified as described above.

### Statistical analysis of cold tolerance experiments

The percentage of survivorship per cavity was calculated for all CT treatments and negative controls (NC). The survivorship of eggs exposed to 25 °C (NC) was analyzed for normality of residuals (D’Agostino & Pearson) and for differences between NCs (unpaired t-test). All NCs passed the normality test except one out of the two *Ae. albopictus* controls (sample size was too small). NCs did not differ significantly from each other, and CT treatments were normalized to the mean of NCs. The sub-zero temperature response of *Aedes* eggs over time was analyzed with the nonlinear regression model *[inhibitor] vs normalized response*. After outlier elimination *via* robust regression outlier removal test (CT1: no outliers; CT2: *Ae. aegypti* − 6 °C one outlier; CT2: *Ae. albopictus* − 2 °C one outlier), all data sets passed the normality of residuals test (D’Agostino & Pearson). LT_50_ values (50% lethal time = time when 50% of eggs did not hatch after treatment) were calculated for normalized data and compared, if possible [17]. In addition, the response to − 2 °C or 0 °C exposure for 8 days in the CT2 experiment were compared intra- and interspecifically using a 2-way ANOVA following Tukey’s *post-hoc* test.

Finally, the number of emerged adults per NC and CT treatment that can contribute to spring populations were evaluated. In addition, the percentage of adults belonging to the wrong species (technical error) was analyzed. Moreover, survival of hatched larvae up to adult stage after extreme exposure was analyzed per test temperature (0 °C, − 2 °C and − 6 °C) and compared between species using Kaplan-Meier survival analysis (log-rank (Mantel-Cox) test). By means of Kaplan-Meier estimator, the survival function from hatched L1 larvae (set as 100%) to emerged adults after previous egg exposure to 1, 2, 4 and 8 days to 0 °C (day 6 was excluded because it is not present in *Ae. aegypti*), to 1, 2, 3, 6 and 8 days to − 2 °C (for a better comparison, day 4 was excluded due to technical error for *Ae. aegypti* described in Table 1) as well as 1 and 2 days to − 6 °C, respectively, was calculated. All statistical analyses for CT experiments were conducted using Prism® (Version 7, GraphPad Software Inc., San Diego-CA, USA).

## Results

### Meta-analysis

Articles or studies that matched our inclusion criteria had been published from four countries: USA (15 articles), Japan (3 articles), Germany (3 articles) and Great Britain (1 article). The earliest study was conducted in 1910 and the latest in 2019. Most articles were published from 1980–1996. One of the earliest studies of 1916 was found by checking the summary of Christophers [41]. From the year 2000 onwards, only six articles were published; two of them in 2019.

**Fig. 2:**
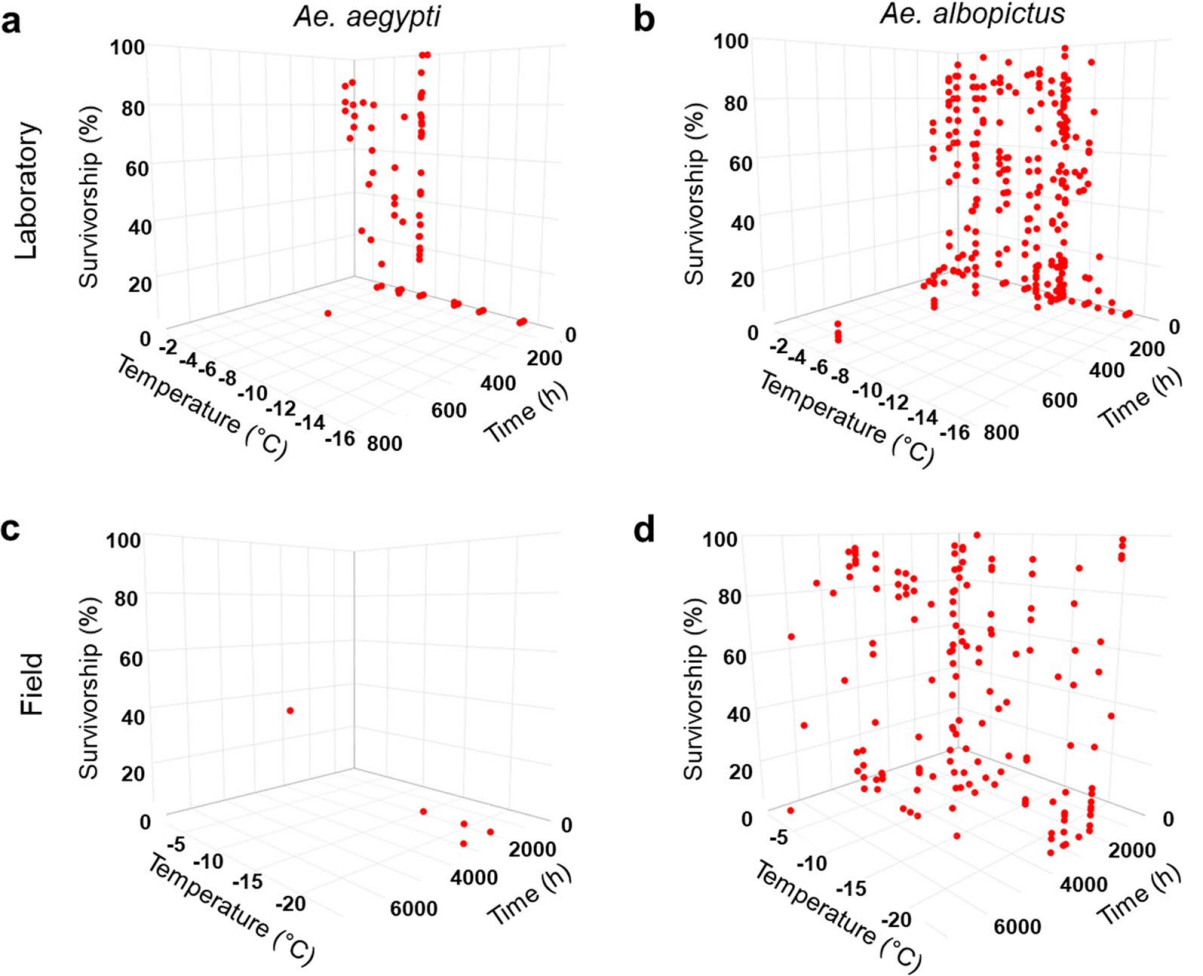
Survivorship (%) of *Ae. aegypti* (**a**, **c**) and *Ae. albopictus* (**b**, **d**) after exposure to different temperatures (°C) over time (h) under laboratory (**a**, **b**) or field (**c**, **d**) conditions

In total, 642 relevant data points for temperatures below 0 °C and respective exposure time and survivorship/mortality could be extracted: 89 data points for *Ae. aegypti* and 553 data points for *Ae. albopictus* (Fig. 2). Thereof, 12.6% stem from laboratory studies with *Ae. aegypti*, 56.2% from laboratory studies with *Ae. albopictus*, 1.3% from field studies with *Ae. aegypti*, and 29.9% from field studies with *Ae. albopictus*. In total, ten different *Ae. aegypti* populations (with three populations whose exact origin is unclear) and 41 different populations of *Ae. albopictus* were tested (Fig. 1, unclear population origins not shown).

For both species, temperatures > − 15 °C were more often examined in laboratory studies, whereas temperatures < − 15 °C were more often examined in field studies (Table 2). The mean test temperatures for *Ae. aegypti* eggs in the laboratory were − 6.2 ± 3.9 °C for 32.6 ± 76.4 h, and for *Ae. albopictus* eggs − 7.7 ± 4.0 °C for 84.1 ± 202.4 h. Most laboratory studies applied short exposure times below 24 h. The field-tested mean temperature and exposure time was − 17.0 ± 6.6 °C for 2118 ± 468.3 h (12.6 ± 2.8 weeks) for *Ae. aegypti,* and − 10.8 ± 7.6 °C for 2640.6 ± 1351.4 h (15.7 ± 8.0 weeks) for *Ae. albopictus*.

**Table 2:** The distribution of reported data points on sub-zero temperature response of *Ae. aegypti* and *Ae. albopictus* at different temperature levels and time points (in percentage)

Parameter	*Ae. aegypti*		*Ae. albopictus*	
	Laboratory	Field	Laboratory	Field
Temperature (°C)				
< − 15	0.8	1.1	2.3	11.1
− 10 to − 15	1.9	0	26.8	2.8
− 5 to − 10	6.7	0	16.8	7.5
0 to − 5	3.3	0.2	10.3	8.6
Exposure time (h)				
24 h	10.1	0	35.4	0
48 h	1.7	0	8.1	0
72 h	0.2	0	3.1	0
96 h	0	0	0.3	0
300 h	0	0	5.5	0.2
1000 h	0.6	0	3.1	2.2
3000 h	0	1.3	0.8	17.1
7000 h	0	0	0	10.4

*Notes*: The proportion of data points tested at different temperature levels (in total 100%) and time points (in total 100%) are shown for each species and type of study (laboratory, field)

For *Ae. aegypti*, even longer exposure times of 360 h (− 5 °C, laboratory), 1848 h (− 15 °C, − 19.4 °C and − 22 °C, field) and 2880 h (− 22 °C, field) were examined, but *Ae. aegypti* eggs could not survive under these conditions [20, 30]. Only in one field experiment, survivorship of *Ae. aegypti* eggs was observed after exposure to − 1.1 °C for 2976 h [35]. Thus, the ability of *Ae. aegypti* eggs for overwintering at sub-zero temperatures in the field has been experimentally proven.

On average, *Ae. albopictus* eggs survived lower sub-zero temperatures with longer exposure times than *Ae. aegypti* eggs. For example, in the laboratory, 47% survivorship of temperate non-diapausing *Ae. albopictus* eggs was observed after exposure to − 10 °C for 144 h [27], or 88% survivorship of tropical non-diapausing *Ae. albopictus* eggs after exposure to − 10 °C for 24 h [29]. *Aedes albopictus* larvae still hatched from non-diapausing conditioned eggs after exposure to 0 °C to − 13 °C for 1 h to 720 h [17, 20–22, 26, 27, 29]. Eggs of *Ae. albopictus* with induced diapause showed survivorship after exposure to 0 °C to − 14 °C for 1–144 h [22, 26, 27]. In the field, diapausing eggs of *Ae. albopictus* showed survivorship after exposure to − 1 °C to − 22 °C for 600 h to 5472 h [15, 18, 19, 24, 25, 28, 30], whereas non-diapausing eggs showed survivorship after exposure to 0 °C to − 22 °C for 336 h to 6576 h [15, 23, 30].

After sub-zero exposure, eggs were reared to adulthood only in one study published in 1938 [31]. However, the latter had to be excluded from our meta-analysis because the survivorship was not reported in numbers, and thus the data could not be analyzed.

Differences between the species become apparent when studying the parameters representing the experimental designs of previous studies (Table 3): the two mosquito species had been kept and bred for different periods of time in the laboratory before they were used in the reported experiments; only 3.2% of the tested *Ae. aegypti* belonged to the < 20th laboratory-bred generation whereas 50.5% of the tested *Ae. albopictus* belonged to the < 20th laboratory-bred generation. Per data point, 173.7 ± 784.7 eggs were used in *Ae. aegypti* studies and 197.2 ± 235.6 eggs in *Ae. albopictus* studies. For both species, replicates (*Ae. aegypti*: 11.5%; *Ae. albopictus*: 53.7%) and controls (*Ae. aegypti*: 5.8%; *Ae. albopictus*: 36.1%) were absent in most of the published experiments. In some studies, eggs had been pre-exposed to different temperatures or different levels of relative humidity. This may have influenced survivorship. For example, *Ae. aegypti* were acclimated before sub-zero exposure in 3.1% of all extracted data points and *Ae. albopictus* in 30.5%, respectively. After (sub)zero exposure, 12.9% of all extracted data points on *Ae. aegypti* eggs reared individuals only to larval stage and 0.2% to pupal stage. In contrast, 73.7% of all extracted data points of *Ae. albopictus* eggs were reared to larval stage and 12.5% to pupal stage. Larvae of either species were never reared to adulthood in any of the included studies.

**Table 3:** The distribution of reported data points on sub-zero temperature response of *Ae. aegypti* and *Ae. albopictus* at different analyzed parameters (in percentage)

Parameter	*Ae. aegypti*	*Ae. albopictus*
Generations		
< 2	1.1	6.1
< 5	0.2	31.9
< 10	0.0	10.6
< 20	1.9	1.9
> 20	0.6	4.7
Unknown	10.1	31.0
Replicates		
Presence	2.3	29.0
Absence	11.5	53.7
Unknown	0	3.4
Controls		
Presence	8.1	37.5
Absence	5.8	36.1
Unknown	0	12.5
Acclimation before sub-zero exposure		
Presence	3.1	30.5
Absence	10.8	55.6
Bleaching of eggs after sub-zero experiment		
Presence	3.3	48.8
Absence	10.6	34.6
Unknown	0	2.8
Rearing to… (after sub-zero exposure)		
Larvae	12.9	73.7
Pupae	0.2	12.5
Adults	0	0
Unknown	0.8	0

*Notes*: The proportion of data points tested at analyzed parameters (each parameter in total 100%) are shown for each species. Analysis parameters are number of generations, absence or presence of replicates, controls, acclimation to different temperatures before sub-zero exposure, bleaching of unhatched eggs after sub-zero experiment, and if larvae were further reared to pupae or adults after sub-zero exposure

### Cold tolerance experiments

**Fig. 3:**
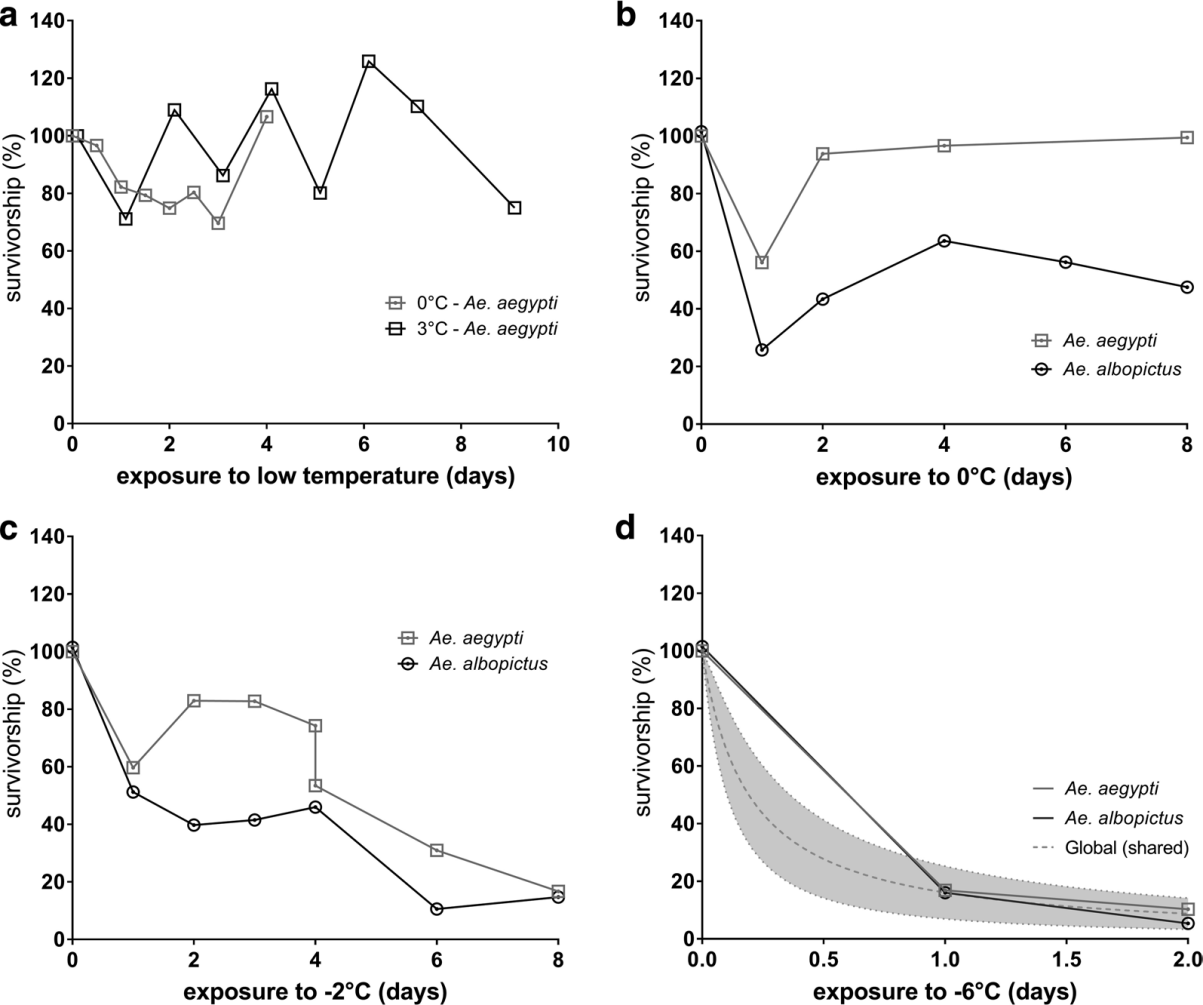
Cold tolerance (CT) of *Ae. aegypti* and *Ae. albopictus* eggs. Mean survivorship (normalized to 25 °C controls, in %) after exposure of *Ae. aegypti* eggs to 0 °C and 3 °C for a maximum of 9 days in experiment CT1 (**a**), and eggs of both species to 0 °C (**b**), − 2 °C (**c**) and − 6 °C (**d**) for a maximum of 8 days in experiment CT2. In addition, the global (shared) CT model is shown (**d**). Mean unnormalized survivorship of controls CT1: 47.8–51.1%; CT2: *Ae. aegypti*: 50.5–51.3%; *Ae. albopictus*: 52.7–53.6% (raw data of treatments in Additional file 2: Table S1)

The CT1 experiment revealed that 3 °C did not have a high influence on the survivorship of *Ae. aegypti* eggs during the exposure times tested (Fig. 3a). Survivorship of *Ae. aegypti* eggs at 0 °C still occurred after 4 days (Fig. 3a, Table 4).

**Table 4:** LT_50_ values and 95% confidence intervals (CI) of *[inhibitor] vs normalized response* models for all data sets

Experiment	CT1		CT2					
Species	*Ae. aegypti*		*Ae. aegypti*	*Ae. albopictus*	*Ae. aegypti*	*Ae. albopictus*	*Ae. aegypti*	*Ae. albopictus*
Egg exposure to	0 °C	3 °C	0 °C	0 °C	− 2 °C	− 2 °C	− 6 °C	− 6 °C
*R*^2^	0.003	− 0.002	− 0.04	0.09	0.33	0.61	0.814	0.79
LT_50_	12.1	371.0	94.8	3.1	4.9	1.4	0.2	0.2
95% Cl of LT_50_	5.8–57.5	39.2–∞	17.9–∞	1.5–6.0	3.4–7.1	0.93–2.1	0.1–0.4	0.007–0.4

*Abbreviations*: CT1, cold tolerance experiment number 1; CT2, cold tolerance experiment number 2

*Notes:* Comparison of LT_50_ of *[inhibitor] vs normalized response* model shows no interspecific significant differences over the exposure time of 2 days at − 6°C (P:0.77)

In general, the survivorship in CT1 (*Ae. aegypti*) and CT2 (both species) experiments showed high variance after egg exposure to sub-zero temperatures as indicated by low regression coefficients and restricted confidence intervals (Table 4). The regression coefficients and results of replicates test indicate that the *[inhibitor] vs normalized response* model adequately describes the CT2 − 6 °C datasets for both species and the CT2 − 2 °C dataset for *Ae. albopictus*.

In experiment CT2, the factor temperature caused a total variation of egg survivorship of 36.0% (*F*_(1, 28)_ = 20.5, *P* = 0.0001), the factor species 7.8% (*F*_(1, 28_) = 4.5, *P* = 0.0440), and their interactions 6.7% (F_(1, 28)_ = 3.8, *P* = 0.0606), respectively. The responses of *Ae. albopictus* eggs to 0 °C and − 2 °C exposure for 8 days were not significantly different. In contrast, the responses of *Ae. aegypti* eggs to (sub)zero temperature exposure for 8 days differed significantly (2-way ANOVA following Tukey’s *post-hoc* test: *P* = 0.0003). The eggs of *Ae. aegypti* were more tolerant to 0 °C and − 2 °C exposure for 8 days if compared to *Ae. albopictus* eggs (Fig. 3b, c). At day 8, interspecific differences became apparent in 0 °C treatments (2-way ANOVA following Tukey’s *post-hoc* test: *P* = 0.0353), but significant interspecific differences vanished in − 2 °C treatments. After 8 days of exposure to 0 °C, the survivorship of both species was higher (*Ae. aegypti*: 99.4%; *Ae. albopictus*: 47.5%) than after 8 days exposure to − 2 °C (*Ae. aegypti*: 16.7%; *Ae. albopictus*: 14.7%; Fig. 3c).

In both species, the survivorship of eggs was low after exposure to − 6 °C for 2 days and then did not differ between species (Table 4). However, survivorship at − 6 °C after 2 days still occurred in both species (*Ae. aegypti* = 10.3%; *Ae. albopictus* = 5.4%; Fig. 3d).

**Fig. 4:**
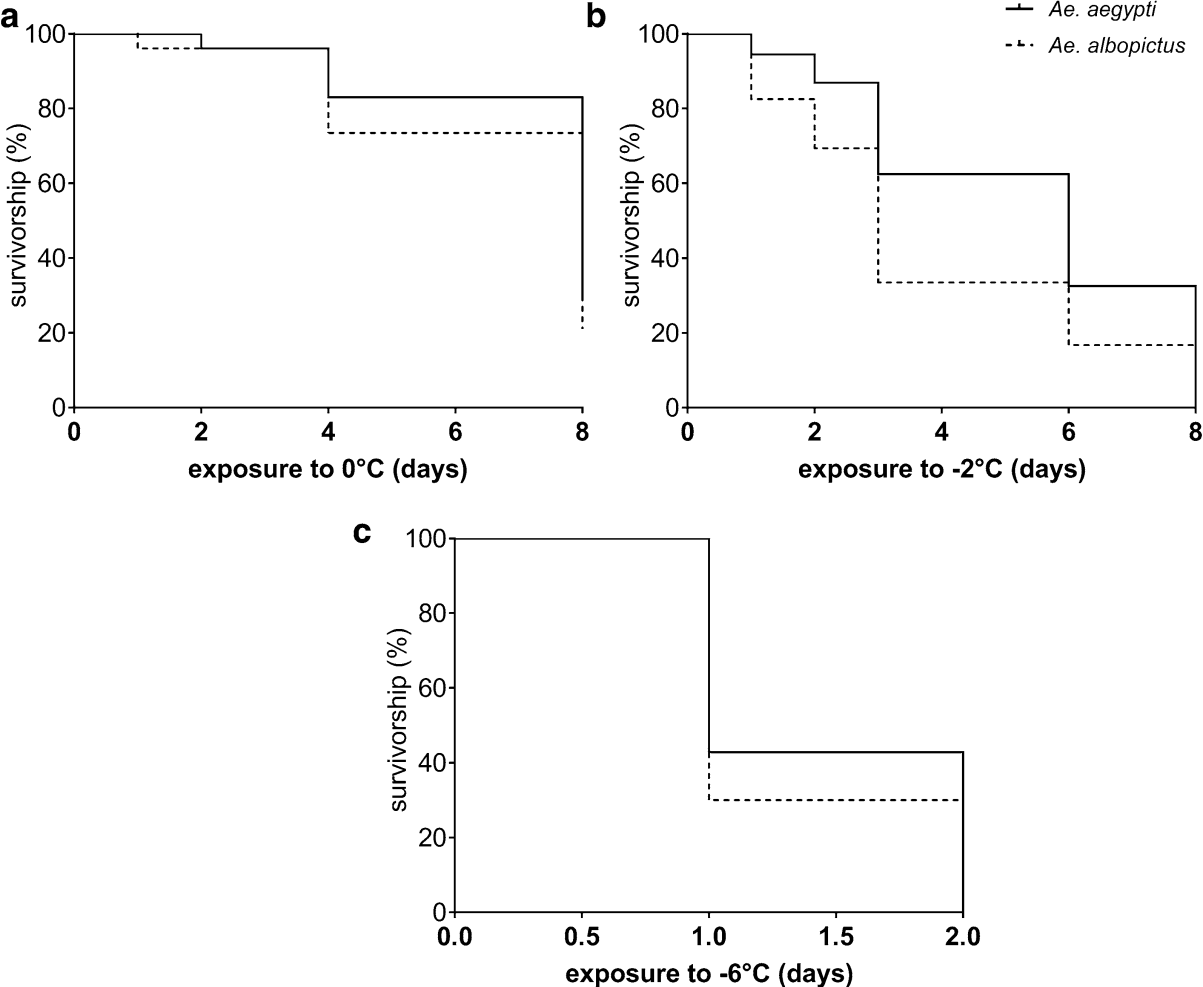
Kaplan-Meier survival analysis of hatched larvae survival up to adult stage after cold exposure of eggs of *Ae. albopictus* and *Ae. aegypti* at 0 °C (**a**), − 2 °C (**b**) and − 6 °C (**c**) over exposure time

*Ae. aegypti* and *Ae. albopictus* hatched after exposure to − 2 °C for 8 days and − 6 °C for 2 days, not all larvae developed until adult stage. In total, 301 adults hatched in the *Ae. aegypti* experiment and 169 adults in the *Ae. albopictus* experiment emerged from eggs after zero and sub-zero temperatures in our experiments. Among these, three *Ae. aegypti* adults emerged after a 6-day exposure to − 2 °C, and one *Ae. albopictus* adult emerged after a 1-day exposure to − 6 °C (detailed description of adult emergence in Additional file 2: Table S6). Estimated survival functions of hatched larvae to emerged adults over exposure time of up to 8 days at − 2 °C significantly differ (log-rank(Mantel-Cox)test; *df* = 1, *P* = 0.0006) between *Ae. aegypti* (50% reduction of survival: 6 days) and *Ae. albopictus* (50% reduction of survival: 3 days) with *Ae. aegypti* showing higher survival of adults (Fig. 4).

Routinely performed microscopical examination of the morphological characters of all emerged mosquitoes in this study revealed that 4.98% out of the 301 emerged *Ae. aegypti* were in fact *Ae. albopictus*, and 12.4% of the 169 adult *Ae. albopictus* were actually *Ae. aegypti*. This highlights the importance of separate verification of taxonomic identity in studies of this type, and mosquito breeding in general. In this case we believe that misidentification during the rearing procedure after sub-zero exposure was the most likely source of error, but contamination during the filling of eggs in tubes (due to electrostatic effects) cannot be excluded.

## Discussion

The merged bibliometric and experimental CT information clearly implies that cold winter temperature is not the limiting factor for the re-establishment of the dengue vector *Ae. aegypti* in southern Europe. Although the meta-analysis shows that the CT of *Ae. aegypti* eggs is underexplored if compared to *Ae. albopictus*, our new experimental data support the literature data. After an 8-day exposure of subtropical eggs from South Asia to 0 °C, interspecific differences in survivorship are present, but these vanish after 8 days of exposure to − 2 °C or 2 days of exposure to − 6 °C.

### Interspecific comparison of cold tolerance

Our experimental CT results of South Asian populations are comparable with those of other laboratory studies. For instance, *Ae. aegypti* eggs of unknown Asian origin were exposed to 0 °C and − 2 °C for up to 24 h [22], *Ae. aegypti* eggs from Polewali (Sulawesi) and Makassar (Sulawesi) to 0 °C for up to 48 h [20], and *Ae. aegypti* eggs from Freetown (Sierra Leone) to − 1.4 °C for up to 24 h and to − 2.2 °C for up to 24 h as well as 72 h [32]. In each of these studies, the survivorship after low/sub-zero temperature exposure was demonstrated. An African population tested at − 2.2 °C for three days showed a survivorship of 25% [32], whereas the normalized mean survivorship in our CT study was 82.8% after egg exposure to − 2 °C for three days. Thus, survival of *Ae. aegypti* differs between populations after sub-zero exposures. In the present study, significant intraspecific differences were detected in *Ae. aegypti* eggs after 8 days of exposure to 0 °C *versus* − 2 °C. Another study showed a survivorship of 65% of *Ae. aegypti* eggs after exposure to 0 °C for 24 h and 45% after exposure to − 2 °C for 24 h [22]. Similarly, a survivorship of 81% after 24 h of exposure to − 1.4 °C and 20% after 24 h of exposure to − 2.2 °C was reported [32]. Hence, a distinct response to 0 °C *versus* − 2 °C was observed in other studies, too. Our present CT study and one other article published in 1938 [31] are the first to report that *Ae. aegypti* eggs can survive sub-zero temperatures and develop into viable adults. The success rate of *Ae. aegypti* reaching adulthood after 0 °C and − 2 °C exposure dropped with exposure time and was zero after exposure to − 6 °C for one or two days. Accordingly, emergence of adults after sub-zero exposure can contribute to population growth in spring even in colder eco-regions.

In comparison to *Ae. aegypti*, intraspecific differences in *Ae. albopictus* eggs after 8 days of exposure to 0 °C or − 2 °C could not be detected. Non-diapausing *Ae. albopictus* eggs from Rimini, Italy, did not differ in their response to a 24 h exposure to 0 °C or − 2 °C, but *Ae. albopictus* eggs from Singapore did [22], as also observed in our study for *Ae. aegypti* from Chitwan, Nepal. Intraspecific variation in cold hardiness between sub-tropical/tropical and temperate populations in *Ae. albopictus* has often been reported [27]. Only one other study [20] evaluated the CT of both species from the same sampling site, although from slightly different laboratory generations (Makassar, *Ae. aegypti*: 15th generation, *Ae. albopictus*: 18th generation; Polewali, *Ae. aegypti*: 17th generation, *Ae. albopictus*: 18th generation). Eggs of *Ae. aegypti* from Makassar, South Sulawesi, Indonesia, showed slightly better survivorship at 0 °C than those of *Ae. albopictus* from the same locality. In the case of mosquitoes from Polewali, West Sulawesi, Indonesia, the survivorship of *Ae. aegypti* eggs at 0 °C was not higher than that of *Ae. albopictus* eggs [20]. In published studies so far, *Ae. albopictus* was only reared to larval or pupal stage after (sub)zero temperature exposures of eggs but never to the adult stage. Thus, to the best of our knowledge, our study is the first to demonstrate the successful development of *Ae. albopictus* into viable adults following sub-zero temperature exposures of eggs. The success rate to reach adulthood after exposure to (sub)zero temperatures was similar in *Ae. aegypti* and *Ae. albopictus* at 0 °C and − 6 °C. At − 2 °C more adults over exposure duration survived and developed within *Ae. aegypti* compared to *Ae. albopictus*, indicating higher CT at − 2 °C in *Ae. aegypti*. However, this result suggests the possibility of both species to establish populations in presence of sub-zero temperatures, which in turn has important epidemiological, prevention and control implications.

### Generation influence on cold tolerance

In order to detect the true CT of field populations, young laboratory generations need to be examined that have the same age as those used in the CT experiments. Accordingly, the first laboratory generation of *Ae. aegypti* eggs was used in our CT1 experiment, whereas eggs of the second laboratory generation of both species were examined in the CT2 experiment. In contrast, the majority of studies included in our meta-analysis evaluated different generations of species and populations and/or used laboratory populations that may have adapted to laboratory conditions [17, 20]. Hoffmann et al. [42] examined laboratory adaptation in insects and stated that an increase/decrease in fitness especially in Diptera in specific trait classes as behavior/reproduction, life history, morphology/size, physiology and stress response was related to laboratory adaptation. In mosquitoes, life history and stress responses seem to be influenced the most [42]. Laboratory adaptation was studied in *Ae. aegypti* for 13 generations using a population size of 100 adults [43]; that study showed that small *Ae. aegypti* populations can suffer a fitness cost. In our CT study, the eggs of the first and second generations were produced by different numbers of adults. Laboratory adaptation can still be assumed to be a minor factor in the present study in view of the fact that eggs from the first and second generations were used. Hence, we expect our results to closely represent actual reality in the field in regard to examined generations.

### High-resolution experimental design

Our meta-analysis revealed differences regarding the study designs (experimental protocols) for the examination of the survivorship of *Ae. aegypti* and *Ae. albopictus*. For instance, hatching media or temperatures differed between studies which may have influenced hatching. Hence, the data extracted from each study may be biased, affecting the comparison of meta-analysis results. In order to minimize effects of laboratory adaptation and consequently artificial biological material effects, a normalization of data to controls is needed to detect true effects. The present CT study is one of a few using controls and replicates, and one of only two studies that normalized the data to the survivorship of the controls [17]. The applied high resolution CT measuring method of Kreß et al. [17] produced reliable results. The negative controls of the second CT generation of *Ae. albopictus* eggs revealed an LT_50_ value of 33.6 h (Table 4, 1.4 * 24 h; after normalization to controls). The first and second generation of *Ae. albopictus* eggs tested by Kreß et al. [17] showed an LT_50_ value of 10.7–37.1 h. Thus, these results from our CT study are congruent with those reported by Kreß et al. [17] for *Ae. albopictus*. Normalization has the advantage of making results more reliable and simplifying the comparison of data. Consequently, future CT studies should normalize data to controls in order to exclude differences in the biological materials used for studies.

Seasonal adaptation in *Ae. aegypti*: The CT of different laboratory generations of both species have been examined by various authors (Additional file 2: Table S1), but in some of those studies it remained unclear when exactly the populations were sampled. In our own CT study, the eggs of *Ae. aegypti* and *Ae. albopictus* were sampled in mid-/end of November which is shortly before the start of winter in Nepal. Hence, a seasonal adaptation and consequently higher CT of the tested egg material, compared to populations sampled in summer, cannot completely be ruled out. So far, there have been no studies regarding a possible seasonal CT adaptation of *Ae. aegypti*. *Ae. aegypti* eggs lack diapause, and the CT shown in our experiments is probably already the maximum that can be achieved by eggs of this lowland Nepal population given the fact survivorship after − 6 °C for 2 days was only 8.59%. In contrast, it is known that eggs of *Ae. albopictus* can enter diapause and show increased CT to sub-zero temperatures after acclimation to lower temperatures before sub-zero exposure. Therefore, they can adapt to colder seasons. A comparison between temperate and tropical strains of *Ae. albopictus* for 24 h showed that diapausing eggs from temperate strains were more cold-hardy than non-diapausing eggs from tropical strains [22]. If *Ae. albopictus* eggs from Chitwan (Nepal) are able to enter diapause, higher survivorship at lower temperatures and over longer sub-zero exposure compared to *Ae. aegypti* could be expected.

A recent study showed a lower hatching response of *Ae. aegypti* eggs from Buenos Aires (Argentina) with a short-day parental photoperiod, and a trend to higher hatching with longer egg storage time in all different photoperiod treatment combinations [44]. These authors suggested that *Ae. aegypti* might have adapted to local climatic conditions which may have or can also cause the evolution of diapause [44]. Thus, experiments on the diapause of *Ae. aegypti* eggs and the CT of these eggs should be conducted, too. In general, the tolerance of all life stages towards lower/sub-zero temperatures should be experimentally tested because a recent study even reported survival of adult *Ae. aegypti* and *Ae. albopictus* after short sub-zero temperature exposures [45].

Moreover, it was shown in *Ae. albopictus* that F1 crosses of a temperate and tropical strain had a higher CT than eggs from an F1 tropical strain [13, 21]. If the females that had produced the eggs came from temperate strains, higher CT was detected in the eggs compared to those from females of tropical strains [13]. Moreover, back crosses of the F1 generation from temperate strains were more cold tolerant then back crosses from tropical strains [13]. Thus, CT can increase if a mixture of populations from different regions occurs. Due to the globalization of trade and travel the latter is a likely scenario, and it could have a huge impact on the future distribution of *Ae. aegypti* as well as *Ae. albopictus*.

### Cold tolerance influenced by origin and season

Our meta-analysis showed that in some other studies (e.g. [20]) the mosquito eggs were cooled down in a stepwise manner until a sub-zero temperature was reached. In our CT experiment, temperature was decreased from 25 °C to sub-zero temperatures in only seven days. Usually, seasonal changes do not happen so rapidly and eggs would have longer acclimation phases to lower temperatures which may even increase CT further as shown by Hanson & Craig [26]. Moreover, in the field fluctuating temperatures and not constant sub-zero temperatures as in the described laboratory experiments are present. However, in a field experiment over 2976 h with a minimum temperature of − 1.1 °C, Hatchett [35] observed that *Ae. aegypti* could survive and hatch even under fluctuating low temperatures. Accordingly, the minimum survival temperatures of eggs summarized in our meta-analysis and CT results should be considered for future vector distribution models as well as risk maps for prevention and control measures, especially for our country of study, Nepal. In order to detect temperature-based distribution limits, the acclimation of eggs prior to cold exposure, the effects of fluctuating temperatures and the ecophysiological plasticity regarding cold temperatures should be further investigated in *Ae. aegypti*. Thus, more populations of *Ae. aegypti* should be experimentally tested to confirm the results described herein. Until now, only ten populations of this species have been evaluated and the present CT study, to our knowledge, is the first to evaluate eggs from a South Asian population. Additionally, the question has to be raised whether populations of *Ae. aegypti* from colder eco-regions may even be more (or less) cold-hardy than the population examined in the present study. It can be assumed that *Ae. aegypti* is likely to establish populations in southern Europe and overwinter especially with regard to climate warming which increases minimum temperatures. To give an example, from Italy, the annual minimum monthly temperature in Modena from 1968–1995 was as low as − 2.4 °C in January and in Foggia as low as 2.9 °C in February, respectively, whereas under climate change scenarios the minimum temperature is expected to be higher [46]. In consequence, in accordance with the presented results, *Ae. aegypti* could probably survive throughout the year and re-establish itself in these regions under climate change scenarios.

## Conclusions

Our analysis shows that the reason why *Ae. aegypti* is presently restricted to subtropical and tropical habitats and lacks permanent populations in especially southern Europe is still unclear. Probably cold winter does not seem to be the limiting factor for this species in southern Europe since *Ae. aegypti* eggs can survive and develop into viable adults after sub-zero temperature exposure up to − 2 °C for 6 days. However, *Ae. aegypti* has been underrepresented in CT testing compared to *Ae. albopictus*. Therefore, future analyses of CT in *Ae. aegypti* should include multiple populations from different eco-regions and address different acclimation scenarios, ecophysiologial plasticity and temperature fluctuations in order to obtain more reliable future distribution models and risk maps of this medically highly important species.

## Supplementary information

**Additional file 1.** PRISMA checklist.

**Additional file 2: Table S1.** Raw data of meta-analysis including raw data of own CT experiment. **Table S2.** Selected data of meta-analysis including raw data of own CT experiments (own data was not included in analysis). **Table S3.** Excluded data of meta-analysis and respective exclusion arguments. **Table S4.** Estimation of exposure time of field studies (meta-analysis). **Table S5.** Estimation of survivorship or mortality from graphs (meta-analysis). **Table S6.** Emergence of adults of CT2 experiment per treatment and species.

## Data Availability

All data generated or analyzed during this study are included in this published article and its additional files.

## Abbreviations

CT: cold tolerance
NC: negative control
CT1: cold tolerance experiment testing 3 °C and 0 °C
CT2: cold tolerance experiment testing 0 °C, − 2 °C and − 6 °C
F1: eggs produced from F0 generation in the laboratory
F2: eggs produced from F1 generation in the laboratory
CI: confidence interval
*R*^2^: coefficient of determination
LT_50_: time when 50% of eggs did not hatch after treatment
*P*: *P*-value
DDT: dichlorodiphenyltrichloroethane

## References

[1] Souza-Neto JA, Powell JR, Bonizzoni M (2019). *Aedes aegypti* vector competence studies: a review. Infect Genet Evol.

[2] Schaffner F, Mathis A (2014). Dengue and dengue vectors in the WHO European region: past, present, and scenarios for the future. Lancet Infect Dis.

[3] Toma L, Di Luca M, Severini F, Boccolini D, Romi R. *Aedes aegypti*: risk of introduction in Italy and strategy to detect the possible re-introduction. Pest Management e salute pubblica. Veterinaria Italiana. Collana di monografie. Monografia 23; 2011. http://www.izs.it/vet_italiana/Collana_di_Monografie/Mon23_2_Toma.pdf.

[4] Almeida APG, Gonçalves YM, Novo MT, Sousa CA, Melim M, Grácio AJS (2007). Vector monitoring of *Aedes aegypti* in the autonomous region of Madeira, Portugal. Euro Surveillance.

[5] Yunicheva YU, Ryabova TE, Markovich NY (2008). First data on the presence of breeding populations of the *Aedes aegypti L.* mosquito in Greater Sochi and various cities of Abkhazia. Meditsinskaia Parazitologiia I Parazitarnye Bolezni.

[6] Scholte EJ, Den Hartog W, Dik M, Schoelitsz B, Brooks M, Schaffner F (2010). Introduction and control of three invasive mosquito species in the Netherlands, July–October 2010. Euro Surveillance.

[7] Caminade C, Medlock JM, Ducheyne E, McIntyre KM, Leach S, Baylis M (2012). Suitability of European climate for the Asian tiger mosquito *Aedes albopictus*: recent trends and future scenarios. J R Soc Interface.

[8] Benedict MQ, Levine RS, Hawley WA, Lounibos LP (2007). Spread of the tiger: global risk of invasion by the mosquito *Aedes albopictus*. Vector-Borne Zoonotic Dis.

[9] European Centre for Disease Preventation and Control (ECDC). Factsheet: *Aedes albopictus*. https://www.ecdc.europa.eu/en/disease-vectors/facts/mosquito-factsheets/aedes-albopictus. Accessed 20 Dec 2019.

[10] European Centre for Disease Prevention and Control (ECDC). *Aedes albopictus*, January 2019. https://ecdc.europa.eu/en/publications-data/aedes-albopictus-current-known-distribution-january-2019. Accessed 10 Dec 2019.

[11] Medlock JM, Hansford KM, Schaffner F, Versteirt V, Hendrickx G, Zeller H (2012). A review of the invasive mosquitoes in Europe: ecology, public health risks, and control options. Vector Borne Zoonotic Dis.

[12] Paupy C (2009). *Aedes albopictus*, an arbovirus vector: from the darkness to the light. Mictobes Infect.

[13] Hanson SM. Cold hardiness of *Aedes albopictus* eggs. Ph.D. Thesis, University of Notre Dame, Notre Dame, IN, USA; 1991.

[14] Hawley AH (1988). The biology of *Aedes albopictus*. J Am Mosq Control Assoc.

[15] Mori A, Oda T, Wada Y (1981). Studies on the egg diapause and overwintering of *Aedes albopictus* in Nagasaki. Trop Med.

[16] Romi R, Severini F, Toma L (2006). Cold acclimation and overwintering of female *Aedes albopictus* in Roma. J Am Mosq Control Assoc.

[17] Kreß A, Oppold AM, Kuch U, Oehlmann J, Müller R (2017). Cold tolerance of the Asian tiger mosquito *Aedes albopictus* and its response to epigenetic alterations. J Insect Physiol.

[18] Jiang Y (2018). Survival of overwintering *Aedes albopictus* eggs under natural conditions in North-Central Florida. J Am Mosq Control Assoc.

[19] Medley KA, Westby KM, Jenkins DG (2019). Rapid local adaptation to northern winters in the invasive Asian tiger mosquito *Aedes albopictus*: a moving target. J Appl Ecol.

[20] Mogi M (2011). Variation in cold hardiness of nondiapausing eggs of nine *Aedes* (*Stegomyia*) species (Diptera: Culicidae) from eastern Asia and Pacific Islands ranging from the tropics to the cool-temperate zone. J Med Entomol.

[21] Tadano T (1990). Cold-hardiness of eggs of the mosquito *Aedes albopictus* (Skuse): among Malaysian and Japanese strains. Trop Biomed.

[22] Thomas S, Obermayr U, Fischer D, Kreyling J, Beierkuhnlein C (2012). Low-temperature threshold for egg survival of a post-diapause and non-diapause European aedine strain, *Aedes albopictus* (Diptera: Culicidae). Parasites Vectors.

[23] Tippelt L, Werner D, Kampen H (2019). Tolerance of three *Aedes albopictus* strains (Diptera: Culicidae) from different geographical origins towards winter temperatures under field conditions in northern Germany. PLoS ONE.

[24] Hanson SM (1995). Field overwinter survivorship of *Aedes albopictus* eggs in Japan. J Am Mosq Control Assoc.

[25] Hanson SM, Mutebi JP, Craig GB, Novak RJ (1993). Reducing the overwintering ability of *Aedes albopictus* by male release. J Am Mosq Control Assoc.

[26] Hanson SM, Craig JRG (1995). Relationship between cold hardiness and supercooling point in *Aedes albopictus* eggs. J Am Mosq Control Assoc.

[27] Hanson SM, Craig GB (1994). Cold acclimation, diapause, and geographic origin affect cold hardiness in eggs of *Aedes albopictus* (Diptera: Culicidae). J Med Entomol.

[28] Hanson SM, Craig GB (1995). *Aedes albopictus* (Diptera: Culicidae) eggs: field survivorship during northern Indiana winters. J Med Entomol.

[29] Hawley W, Reiter P, Copeland R, Pumpuni C, Craig GJ (1987). *Aedes albopictus* in North America: probable introduction in used tires from northern Asia. Science.

[30] Hawley WA, Pumpuni CB, Brady RH, Craig GB (1989). Overwintering survival of *Aedes albopictus* (Diptera: Culicidae) eggs in Indiana. J Med Entomol.

[31] Rozeboom LE. The overwintering of *Aedes aegypti* L. in stillwater Oklahoma. In: Acad Sci. 1938;81–2.

[32] Bacot AW (1916). Report of the entomological investigation undertaken for the Commission for the year, August 1914- to July 1915. Yellow Fever Comm.

[33] Bond HA, Keirans JE, Merle FB (1970). Environmental influences on the viability of overwintering *Aedes aegypti* (L.) eggs. Mosq News.

[34] Davis NC (1931). The effects of heat and of cold upon *Aedes* (*Stegomyia*) *aegypti*. Am J Epidemiol.

[35] Hatchett SP (1946). Winter survival of *Aedes aegypti* (L.) in Houston, Texas. Public Health Rep.

[36] Reed W, Owen M. Yellow fever—a compilation of various publications. In: Owen M, editor. Washington: Washington Government Printing Office; 1911.

[37] Craig GBJ, Hawley WA. The Asian tiger mosquito, *Aedes albopictus*: whither, whence, and why not in Virginia? In: Centennial of Entomology at Virginia Polytechnic University, Blacksburg, 6–10 September 1991.

[38] RStudio Team. RStudio: integrated development for R. RStudio, Inc., Boston, MA. 2015. http://www.rstudio.com/. Accessed 10 Nov 2019.

[39] Kreß A, Kuch U, Oehlmann J, Müller R (2014). Impact of temperature and nutrition on the toxicity of the insecticide λ-cyhalothrin in full-lifecycle tests with the target mosquito species *Aedes albopictus* and *Culex pipiens*. J Pest Sci.

[40] Müller R, Knautz T, Volker J, Kreb A, Kuch U, Oehlmann J (2013). Appropriate larval food quality and quantity for *Aedes albopictus* (Diptera: Culicidae). J Med Entomol.

[41] Christophers RS (1960). *Aedes aegypti* (L.) the yellow fever mosquito—its life history, bionomics and structure. Regulatory risk and the cost of capital: determinants and implications for rate regulation.

[42] Hoffmann AA, Ross PA (2018). Rates and patterns of laboratory adaptation in (mostly) insects. J Econ Entomol.

[43] Ross PA, Endersby-Harshman NM, Hoffmann AA (2019). A comprehensive assessment of inbreeding and laboratory adaptation in *Aedes aegypti* mosquitoes. Evol Appl.

[44] Fischer S, De Majo MS, Di Battista CM, Montini P, Loetti V, Campos RE (2019). Adaptation to temperate climates: evidence of photoperiod-induced embryonic dormancy in *Aedes aegypti* in South America. J Insect Physiol.

[45] Zhang D, Xi Z, Fan Y, Zheng X, Li Y, Wu Y (2019). Water-induced strong protection against acute exposure to low subzero temperature of adult *Aedes albopictus*. PLoS Negl Trop Dis.

[46] Tubiello FN, Donatelli M, Rosenzweig C, Stockle CO (2000). Effects of climate change and elevated CO_2_ on cropping systems: model predictions at two Italian locations. Eur J Agron.

[47] Kraemer MUG, Sinka ME, Duda KA, Mylne AQN, Shearer FM, Barker CM (2015). The global distribution of the arbovirus vectors *Aedes aegypti* and *Ae. albopictus*. Elife.

